# Characterizing sleep-related complaints in head and neck cancer survivors with tumors of airway relevance following radiotherapy

**DOI:** 10.1016/j.ctro.2026.101248

**Published:** 2026-07-29

**Authors:** Alberto Herrero Babiloni, Christina Minville, Pierre Rompre, Nelly Huynh, Félix P. Nguyen-Tan, Edith Filion, Houda Bahig, Cibele Dal Fabbro, Eric Dufresne, Matthieu Schmittbuhl, Gilles Lavigne

**Affiliations:** a Department of Stomatology, Centre hospitalier de l'Université de Montréal (CHUM), Montréal, QC, Canada; b Faculté de Médecine dentaire, Université de Montréal, Montréal, QC, Canada; c Department of Radiology, Centre Hospitalier de l'Université de Montréal (CHUM), University of Montreal, Montréal, QC, Canada; d Centre de recherche de l'institut universitaire de gériatrie de Montréal, Montréal, Canada; e Faculty of Dental Medicine and Oral Health Sciences, Faculty of Medicine - Neurology and Neurosurgery, McGill University, Montreal, Canada

## Abstract

• Sleep complaints were highly prevalent after radiotherapy for HNC survivors.
• High OSA risk was 3.3-fold greater than population reference values.
• Poor sleep quality was common, with 69% scoring above the PSQI cutoff.
• More than half of participants reported clinically significant fatigue.
• Sleep issues did not differ by tumor site, airway impact or concurrent chemotherapy.

## Introduction

Sleep disturbances are increasingly being recognized as a problem in patients treated for head and neck cancers (HNC) [1], [2]. This is particularly relevant in cancers involving upper-airway structures, including oropharyngeal cancer (OPC), where both the tumor itself and its treatment may affect airway anatomy and function. However, the existing literature remains scarce and fragmented, in part because studies often combine different tumor sites and treatment modalities under the broad HNC category [1], [2]. This heterogeneity, spanning surgical, chemoradiation, and radiotherapy approaches, complicates the interpretation of sleep-related symptoms and limits more precise clinical characterization. In tumors affecting the oropharyngeal and related upper-airway regions, radiotherapy may further contribute to changes in airway patency and surrounding tissues, potentially increasing the risk of obstructive sleep apnea (OSA) and other sleep-related complaints [3]. Furthermore, differences in anatomical involvement and treatment exposure, including the use of chemotherapy, may contribute to variability in the type and severity of sleep-related symptoms across HNC survivors [1], [4]. Indeed, although radiotherapy is more directly implicated in structural upper-airway changes, chemotherapy may also influence sleep-related symptom burden through broader effects on respiratory function and muscle strength, and general treatment toxicity [5], [6]. Accordingly, a more focused characterization of sleep symptoms in radiotherapy-treated HNC survivors, particularly those with tumors involving airway-relevant regions across clinically relevant disease- and treatment-related subgroups, may help clarify the nature and magnitude of this burden and better inform screening and survivorship care. Importantly, beyond prevalence estimates, quantifying how these symptoms compare to normative population values obtained from larger studies provides a clearer perspective on the excess burden attributable to cancer and its treatment [7]. The objective of this study was to characterize sleep-related complaints, including snoring, OSA risk, excessive daytime sleepiness, insomnia symptoms, sleep quality, and fatigue, in a cohort of head and neck cancer survivors with tumors of airway relevance in a tertiary oncology center. Additionally, we explored whether these complaints differed according to clinically relevant disease- and treatment-related characteristics, particularly anatomical involvement and chemotherapy exposure. This work represents the first phase of a larger clinical research initiative aimed at improving the recognition and management of sleep disturbances in this population (NCT05719779).

## Methods

Recruitment was conducted through printed flyers and direct invitation. In total, 200 flyers were distributed across the stomatology, radio-oncology, and pulmonology departments at the Centre hospitalier de l'Université de Montréal (CHUM). The survey platform logged 91 entries, of which 15 were duplicates and 16 were incomplete (i.e., only partial questionnaires). For duplicate entries (i.e., multiple submissions from the same individual at different times), the first entry of each participant was retained. Moreover, two additional participants were excluded because: a) their radiotherapy treatment was on hold and had not yet resumed at the time of study participation; b) although affecting the maxilla, the cancer was an oligodendroglioma located in the brain (inferior parietal region). This left 58 unique participants with adequately completed questionnaires (≈30 min) for inclusion in the analyses. All participants had previously undergone curative-intent radiotherapy for HNC at CHUM. This cross-sectional study received institutional ethics approval (protocol #21–209), and participants provided online informed consent. Eligibility criteria included age ≥ 18 years, prior completion of curative-intent radiotherapy for HNC, and the ability to complete online questionnaires in French or English.

Participants completed web-based questionnaires collecting sociodemographic and clinical information (e.g., age, sex, body mass index [BMI], education, employment, marital status, and income). Additionally, the Hospital Anxiety and Depression Scale (HADS, range 0–21) [8] and the 12-item Short Form Health Survey (SF-12, norm-based score) were completed for quality of life [9]. For sleep and fatigue variables, the STOP-Bang questionnaire (range 0–8) [10], Epworth Sleepiness Scale (ESS, range 0–24) [11], Insomnia Severity Index (ISI, range 0–28) [12], Pittsburgh Sleep Quality Index (PSQI, range 0–21) [13], and Chalder Fatigue Scale (CFS, range 0–33) [14], were used. Although snoring is included as one item within the STOP-Bang questionnaire, it was also described separately because STOP-Bang was used as a composite OSA-risk screening tool, whereas snoring was considered a symptom-level sleep complaint. This distinction was considered clinically relevant because STOP-Bang integrates symptom items with demographic and anthropometric risk factors, including age, sex, BMI, and neck circumference, while snoring captures a specific upper-airway symptom. Snoring and OSA-related complaints are among the sleep-related concerns frequently described in individuals with head and neck cancer [1], and snoring may also affect bed-partner sleep [15]. Therefore, snoring was reported separately to distinguish the prevalence of this specific symptom from the broader composite OSA-risk classification. Additional cancer- and treatment-related variables, including anatomical involvement and chemotherapy exposure, were obtained from clinical records/database and were used in exploratory subgroup comparisons. Primary location was summarized descriptively, whereas anatomic group was categorized into the following descriptive groups: thyroid, hypopharynx, larynx, lymph nodes, oral cavity, oropharynx, salivary glands, and sinonasal. For exploratory inferential analyses, anatomic group was further collapsed into a binary variable contrasting oropharynx versus other sites. In addition, a clinically derived airway-impact grouping was used to classify participants into low-airway-impact versus high-airway-impact categories, based on the presumed relative potential of the tumor site to affect upper-airway structure or respiration [16]. Thus, high-airway-impact included the oropharynx, tonsil, tonsillar fossa, base of tongue, nasopharynx, and hypopharynx, whereas low-airway-impact sites included salivary, oral cavity, sinonasal, thyroid, or other non-pharyngeal locations if any. Chemotherapy exposure was coded dichotomously as yes/no. Radiotherapy dose and number of fractions were collected and summarized descriptively only because of limited variability across participants.

Standard scoring procedures were applied for each instrument, with recommended cut-offs used to classify symptom severity, those being: STOP-Bang ≥5 indicating high OSA risk (3–4 intermediate, 0–2 low) [10], ESS 0–7 unlikely, 8–9 average, 10–15 excessive, and 16–24 severe daytime sleepiness [11], ISI 0–7 absence, 8–14 mild, 15–21 moderate, and 22–28 severe insomnia [12], PSQI >5 indicating poor sleep quality [13], and CFS ≥ 18 indicating elevated fatigue [14]. HADS anxiety and depression subscale scores were interpreted as 0–7 normal, 8–10 borderline, and 11–21 abnormal symptoms [8], while SF-12 physical and mental component summary scores were interpreted as norm-based scores, with a reference population mean of 50 and standard deviation of 10 [9], where higher scores indicate better health status. However, as HADS and SF-12 were not primary sleep-related outcomes these interpretive values were provided only to contextualize the descriptive results and were not used to classify participants or define analytical outcomes. To place our results in perspective with existing literature, general population reference values were drawn from published external normative datasets for each instrument. Differences between the HNC cohort and population norms were expressed as mean or risk differences with 95% confidence intervals (CI), and standardized effect sizes (Cohen's d for continuous outcomes; odds ratios [OR] for categorical outcomes).

In addition, exploratory subgroup comparisons were conducted to examine whether sleep-related outcomes differed according to clinically relevant disease- and treatment-related characteristics, particularly anatomic group (oropharynx vs other), airway-impact grouping (high vs low), and chemotherapy exposure (yes vs no). Group differences in continuous outcomes were assessed using independent-samples *t*-tests. Levene's test was used to assess variance homogeneity prior to each comparison. Welch's correction was applied where variances were unequal. Normality was evaluated using Shapiro-Wilk tests, and Mann-Whitney *U* tests were conducted as a sensitivity check where the assumption was violated. Effect sizes are reported as Cohen's d, corrected for small-sample bias (Hedges' correction). For the binary outcomes, Fisher's Exact test was used, with proportion differences and 95% CIs estimated via the Agresti-Coull method. The odds ratio was estimated by conditional maximum likelihood. Statistical significance was set at *p* < 0.05. Because of the modest sample size and the limited number of participants within some subgroup and outcome categories, multivariable models adjusted for age, sex, BMI, or other covariates were not performed to avoid unstable estimates and overfitting. Accordingly, subgroup analyses were considered exploratory.

## Results

Sociodemographic characteristics can be observed in Table 1. Participants were predominantly male (79.3%) and had a mean age of 56.6 ± 11.8 years (range: 22–79). A majority held a university degree (53.4%), and most were either employed full-time (60.3%) or retired (25.9%). Regarding cultural background, 84.5% self-identified as North American. Mean body mass index (BMI) was 26.1 ± 5.5 kg/m^2^. Anxiety and depression scores on the Hospital Anxiety and Depression Scale averaged 5.7 ± 3.3 and 5.0 ± 3.7, respectively, and were within the normal range. However, aggregate SF-12 scores reflected a moderate symptom burden on quality of life, with a mean physical health score of 45.5 ± 9.6 and mental health score of 43.6 ± 9.0. Regarding clinical and treatment characteristics, most participants had squamous cell carcinoma (77.6%). The most common primary location by anatomic group was the oropharynx (58.6%). The remaining tumors were distributed across salivary glands (12.1%), oral cavity (10.3%), sinonasal sites (10.3%), larynx (3.4%), hypopharynx (1.7%), lymph nodes (1.7%), and thyroid gland (1.7%). Half of the sample had received concurrent chemotherapy (50.0%). Radiotherapy characteristics were relatively homogeneous overall, with most participants receiving 70 Gy (69.0%) delivered in 33 fractions (77.6%).

**Table 1:** Sociodemographic, psychological, and sleep-related variables.

Variable	Mean (SD) or n (%)
Sociodemographic characteristics	
Age (years)	56.6 (11.8)

Sex	
Female	12 (20.7%)
Male	46 (79.3%)

Education	
University	31 (53.4%)
College/CEGEP	10 (17.2%)
High School	17 (29.3%)

Employment	
Full-time	35 (60.3%)
Part-time	5 (8.6%)
Occasional	2 (3.4%)
Student	1 (1.7%)
Retired	15 (25.9%)

Cultural Origin⁎	
North American	49 (84.5%)
South/Central America	1 (1.7%)
Africa	1 (1.7%)
Europe	4 (6.9%)
Asia	2 (3.4%)
Middle East/North Africa	5 (8.6%)

Personal Income	
< $20,000	3 (6.5%)
$20,000–49,999	14 (30.4%)
$50,000–69,999	9 (19.6%)
≥ $70,000	20 (43.5%)

Household Income	
< $30,000	5 (11.1%)
$30,000–69,999	10 (22.2%)
$70,000–109,999	10 (22.2%)
$110,000–139,999	11 (24.4%)
≥ $140,000	9 (20.0%)
BMI (kg/m²)	26.1 (5.5)

Clinical and treatment characteristics	

Cancer type	
Squamous cell carcinoma	45 (77.6%)
Others	13 (22.4%)

Primary location	
Oropharynx	34 (58.6%)
Salivary glands	7 (12.1%)
Oral cavity	6 (10.3%)
Sinonasal	6 (10.3%)
Larynx	2 (3.4%)
Hypopharynx	1 (1.7%)
Lymph nodes	1 (1.7%)
Thyroid	1 (1.7%)

Chemotherapy exposure	
Yes	29 (50.0%)
No	29 (50.0%)

Radiotherapy dose	
20 Gy	1 (1.7%)
30 Gy	2 (3.4%)
60 Gy	9 (15.5%)
66 Gy	6 (10.3%)
70 Gy	40 (69.0%)

Number of fractions	
5	1 (1.7%)
15	2 (3.4%)
30	4 (6.9%)
33	45 (77.6%)
34	1 (1.7%)
35	3 (5.2%)
Undetermined	2 (3.4%)

Psychological and quality-of-life variables	
HADS – Anxiety	5.7 (3.3)
HADS – Depression	5.0 (3.7)
SF-12 Physical Health Score	45.5 (9.6)
SF-12 Mental Health Score	43.6 (9.0)

Sleep and fatigue variables	
Epworth Sleepiness Scale (ESS)	8.3 (4.6)
Insomnia Severity Index (ISI)	9.7 (6.3)
Pittsburgh Sleep Quality Index (PSQI)	8.1 (3.9)
Chalder Fatigue Scale (CFS)	18.3 (5.2)
Snoring (categorical) *If sharing a room, frequency of loud snoring during the last month*	
Not once during the last month	15 (37.5%)
Less than once per week	8 (20.0%)
One or two times per week	5 (12.5%)
Three times per week or more	12 (30.0%)

⁎ Values do not round up to 100% due to participants marking two categories.

**Fig. 1:**
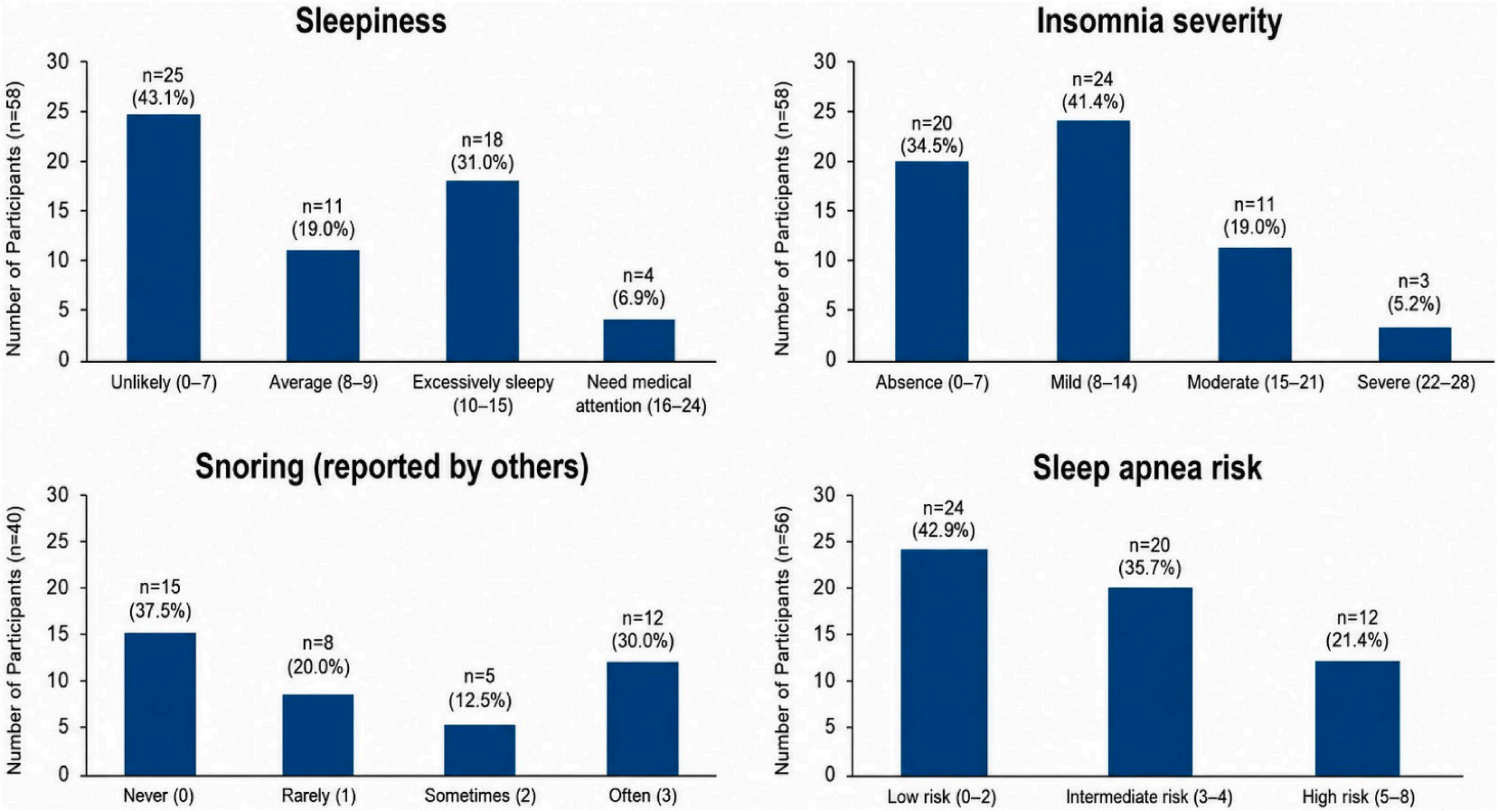
Distribution of sleep-related symptoms and risk factors among participants based on validated screening tools. Legend: Epworth Sleepiness Scale: 0–7 = Unlikely, 8–9 = Average, 10–15 = Excessively sleepy, 16–24 = Need medical attention. Insomnia Severity Index: 0–7 = Absence, 8–14 = Mild, 15–21 = Moderate, 22–28 = Severe. STOP-Bang Questionnaire: 0–2 = Low risk, 3–4 = Intermediate risk, 5–8 = High risk. Snoring (as reported by others): 0 = Never, 1 = Rarely, 2 = Sometimes, 3 = Often.

**Fig. 2:**
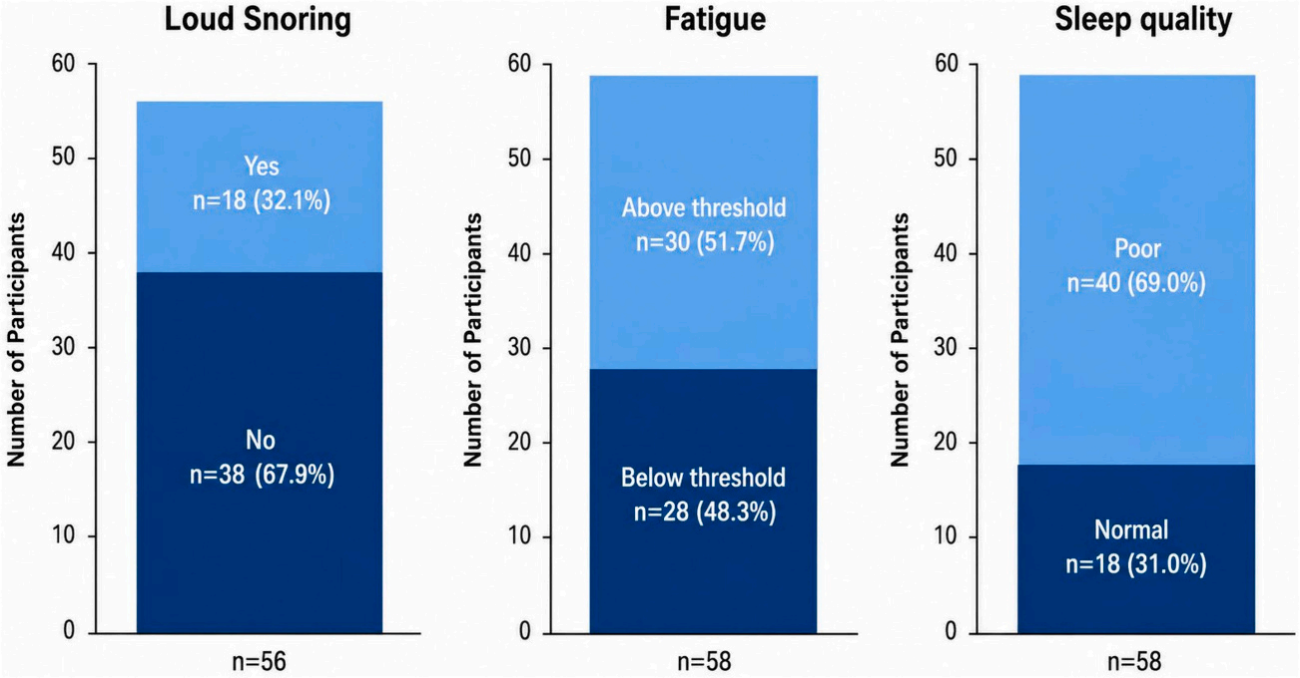
Prevalence of loud snoring, clinically significant fatigue, and poor sleep quality among participants. Legend: Loud Snoring: No = Not reported to snore loudly, Yes = Loud snoring reported (e.g., heard through closed doors or disturbing bed partners). Chalder Fatigue Scale: <18 = Below threshold, ≥18 = Above threshold (clinically significant fatigue). PSQI: ≤5 = Normal sleep quality, >5 = Poor sleep quality.

Sleep complaints were frequently reported. On the STOP-Bang questionnaire, 21.4% of the participants with available data (*n* = 56) were classified as having high risk for OSA, 35.7% as intermediate, and 42.9% as low risk (Fig. 1). Most participants admitted to snoring, with 62.5% reporting some degree of snoring (rarely, sometimes, or often), including 30.0% who indicated they snored often (Fig. 1). Moreover, 32.1% of participants admitted to snoring loudly (i.e., heard through closed doors or noted by a bed partner) (Fig. 2). Daytime sleepiness was also common (ESS mean: 8.3 ± 4.6), with 50% of participants being categorized into the “average” or “excessive” range on the ESS, and 6.9% scored as “severe” sleepiness (Fig. 1). Regarding insomnia, the mean ISI score was 9.7 ± 6.3. Although one-third of the sample reported no insomnia symptoms, 41.4% met criteria for mild, 19.0% for moderate, and 5.2% for severe insomnia (Fig. 1). Overall sleep quality, as measured by the PSQI, was poor, with a mean score of 8.1 ± 3.9. Indeed, most participants (69%) had a PSQI >5, indicating poor sleep quality. Additionally, more than half of the participants (51.7%) reported elevated fatigue, defined by a CFS score of ≥18 (CFS mean: 18.3 ± 5.2).

When placed in the context of population norms, HNC survivors showed markedly greater vulnerability in specific domains (Table 2). High OSA risk was observed in 21.4% of patients compared with 6.7% in community samples [17], corresponding to an odds ratio of 3.3 (95%CI: 1.8 to 6.1). Sleep quality was also substantially impaired, with a mean PSQI score 3.1 points higher than norms (d = 0.91) [18], while fatigue levels were nearly eight points higher on the Chalder scale (d = 1.57) [19], representing a large effect. By contrast, daytime sleepiness was only modestly elevated (mean difference 0.4, d = 0.10) [20], and insomnia severity was essentially comparable to normative values (mean difference − 0.5, d = −0.08) [21]. These comparisons indicate that poor sleep quality, fatigue, and elevated OSA risk are disproportionate complaints in this sample.

**Table 2:** Comparison of sleep and fatigue measures between study sample (*n* = 58) and general population values.

Instrument	Study sample (mean ± SD or %)	General population (mean ± SD or %)	Mean difference /Risk difference (95% CI)	Effect size (Cohen's *d* or OR, 95% CI)
STOP-Bang (OSA high risk)	21.4%	6.7%	14.7% (95% CI 5.0–26.5%)	OR = 3.3 (95% CI 1.8–6.1)
ESS (daytime sleepiness)	8.3 ± 4.6	7.9 ± 3.7	0.4 (95% CI –0.8 to 1.6)	*d* = 0.10 (95% CI -0.19 to 0.38)
ISI (insomnia severity)	9.7 ± 6.3	10.2 ± 6.1	−0.5 (95% CI –2.3 to 1.3)	*d* = −0.08 (95% CI –0.37 to 0.21)
PSQI (sleep quality)	8.1 ± 3.9	5.0 ± 3.4	3.1 (95% CI 2.2 to 4.0)	*d* = 0.91 (95% CI 0.65 to 1.17)
Chalder Fatigue Scale	18.3 ± 5.2	10.6 ± 4.9	7.7 (95% CI 6.4 to 9.0)	*d* = 1.57 (95% CI 1.30 to 1.83)

Values are presented as mean difference (95% confidence interval [CI]) or risk difference (95% CI), alongside standardized effect sizes with corresponding CIs. For continuous outcomes, Cohen's *d* was used to quantify effect size; values of 0.2, 0.5, and 0.8 represent small, medium, and large effects, respectively. For categorical outcomes (STOP-Bang high risk), results are shown as risk difference (RD), and odds ratio (OR) with 95% CIs. Positive mean or risk differences indicate higher values in the study sample compared to the general population.

References used for normative data in order of display: 1. Kang J, Koo HK, Kang HK, et al., Prevalence of high-risk group for obstructive sleep apnea using the STOP-Bang questionnaire and its association with cardiovascular morbidity. Front Neurol. 2024 Dec 9;15:1394345.

2. Sander C, Hegerl U, Wirkner K, et al., Normative values of the Epworth Sleepiness Scale (ESS), derived from a large German sample. Sleep Breath. 2016 Dec;20(4):1337–1345.

3. Dieperink KB, Elnegaard CM, Winther B, et al., Preliminary validation of the insomnia severity index in Danish outpatients with a medical condition*.* J Patient Rep Outcomes. 2020 Mar 2;4(1):18.

4. Hinz A, Glaesmer H, Brähler E, et al., Sleep quality in the general population: psychometric properties of the Pittsburgh Sleep Quality Index, derived from a German community sample of 9284 people*.* Sleep Med. 2017 Feb;30:57–63.

5. Krakau L, Wicke F, Häuser W, et al., Chronic fatigue: psychometric properties and updated norm values of the Chalder fatigue scale in a cross-sectional sample representative of the German population*.* Ann Med. 2025 Dec;57(1):2524087.

Exploratory subgroup comparisons by anatomical site, airway impact, and chemotherapy status are summarized in Supplementary Tables A–C. No statistically significant between-group differences were observed across any comparison. A numerical trend was noted for HADS-Depression scores in the airway-impact grouping, with higher scores in the high-impact group compared to the low-impact group (5.61 ± 3.62 vs. 3.50 ± 3.56; *p* = 0.055).

## Discussion

This study highlights the high prevalence of sleep-related complaints such as snoring, insomnia symptoms, excessive daytime sleepiness, poor sleep quality, and fatigue, among head and neck cancer survivors with tumors of airway relevance. Our findings align closely with previous literature [1], [2], including a recent study among HNC survivors that employed comparable assessment tools [4]. However, their cohort included a broader mix of tumor sites—only 19.9% had OPC—and encompassed a range of treatment modalities, including surgery alone, radiotherapy alone, and combined regimens. In the present study, comparisons with normative population values further suggested that poor sleep quality, fatigue, and elevated OSA risk were the most disproportionate complaints, whereas daytime sleepiness was only modestly elevated and insomnia severity was broadly comparable to normative values. These comparisons should be interpreted cautiously, however, because the normative values were derived from external published datasets rather than from a prospectively recruited matched control group, and the HNC sample may differ from the reference populations in clinically relevant ways.

Radiotherapy, while critical in managing HNC, can introduce a range of anatomical and physiological changes that can increase vulnerability to sleep disturbances [2], [22], [23]. In fact, recent work in OPC survivors has shown that more than two-thirds met diagnostic criteria for OSAS on respiratory polygraphy [3], often in the absence of daytime sleepiness symptoms. Radiotherapy can induce fibrosis and edema, narrowing the airway lumen. Moreover, it can also produce damage to neuromuscular structures and thus impair reflexive airway stability during sleep, contributing to increased collapsibility and snoring. Reduced coordination of pharyngeal musculature further disrupts normal breathing patterns and may elevate the risk of OSA, particularly in the supine position [24]. Beyond structural effects, radiotherapy and the broader cancer-treatment context may also contribute to sleep and circadian rhythm disturbance through multifactorial pathways, including systemic inflammatory responses, altered sleep–wake and activity–rest patterns, and disruption of normal routines during prolonged treatment courses [23]. Notably, melatonin has been examined in cancer and radiotherapy contexts because of its antioxidant, anti-inflammatory, and potential neuroprotective properties, suggesting that melatonin-related pathways may be biologically relevant to treatment-related sleep and circadian disruption [25], [26]. Moreover, radiotherapy frequently damages salivary glands leading to xerostomia [27], which has been linked to disrupted sleep continuity due to frequent nighttime awakenings, oral discomfort, and increased upper airway resistance [28], [29]. Indeed, Pecorari et al. found that xerostomia was the most commonly reported symptom in their sample, especially among those receiving radiotherapy [4].

Fatigue is also a common side effect of radiation therapy for cancer [30]. This can arise from the body's response to radiation, which damages both cancer cells and healthy cells requiring the body to expend energy on repair, from disturbed sleep, or from mental stress attributed to cancer treatment burden [30]. Certainly, mood disorders and other psychological factors often present in these patients can both disrupt sleep and induce fatigue [31], [32], [33]. While HADS scores in our sample did not indicate elevated anxiety or depression on average, the SF-12 mental component scores suggested some degree of psychological burden. Exploratory subgroup analyses did not identify statistically significant differences in sleep-related, psychological, or quality-of-life outcomes according to anatomical group, airway-impact category, or chemotherapy exposure, suggesting that sleep-related complaints after radiotherapy are broadly distributed across clinical subgroups rather than being confined to a single anatomical or treatment-defined category. On the other hand, there is also evidence suggesting that OSA and sleep problems may be associated with HNC and overall cancer development, respectively [16], [34], suggesting a complex relationship.

Several limitations should be considered when interpreting these findings. First, the sample size was modest, which limited statistical power for subgroup comparisons and precluded more extensive adjusted analyses. Second, the cross-sectional design prevents conclusions about temporal or causal relationships between cancer treatment and sleep-related complaints. Third, sleep outcomes were assessed using self-report questionnaires rather than objective sleep measures, such as polysomnography or home sleep apnea testing. Fourth, comparisons with population norms should be interpreted carefully. The reference values were drawn from different published studies, each with its own sample characteristics, and it was therefore not possible to adjust for potential confounders such as age or sex distribution. To the extent that the demographic profile of our sample diverges from those of the reference populations, some of the observed differences may partly reflect these compositional differences rather than true clinical distinctions. Finally, data on chronic comorbidities and total treatment duration in days were not systematically available in the analytic dataset and therefore could not be analyzed. Specifically, precise treatment-course information, including start and end dates, treatment interruptions, delays, or schedule modifications, was not consistently captured. Deriving this variable retrospectively could therefore have introduced misclassification.

Despite these limitations, this survey provides additional characterization of sleep-related complaints in a radiotherapy-treated HNC cohort with tumors of airway relevance. The findings are consistent with prior observations in broader HNC and OPC samples and suggest a high prevalence of poor sleep quality, fatigue, snoring, and elevated OSA risk in this population. These results support the value of longitudinal studies with systematic treatment-course data and objective sleep assessment, as well as more targeted screening to identify patients who may benefit from sleep management during survivorship care.

## CRediT authorship contribution statement

**Alberto Herrero Babiloni:** Writing – original draft, Visualization, Methodology, Investigation, Conceptualization. **Christina Minville:** Writing – review & editing, Investigation, Data curation. **Pierre Rompre:** Writing – review & editing, Validation, Software, Methodology, Formal analysis, Data curation. **Nelly Huynh:** Writing – review & editing, Project administration, Methodology, Investigation, Conceptualization. **Félix P. Nguyen-Tan:** Resources, Investigation, Conceptualization. **Edith Filion:** Resources, Investigation. **Houda Bahig:** Resources, Investigation. **Cibele Dal Fabbro:** Writing – review & editing, Validation, Supervision, Investigation, Conceptualization. **Eric Dufresne:** Supervision, Resources, Investigation. **Matthieu Schmittbuhl:** Writing – review & editing, Supervision, Investigation, Conceptualization. **Gilles Lavigne:** Writing – review & editing, Validation, Supervision, Investigation, Conceptualization.

## Declaration of competing interest

The authors declare that they have no known competing financial interests or personal relationships that could have appeared to influence the work reported in this paper.
