# Supplementary material for: Characterizing sleep-related complaints in head and neck cancer survivors with tumors of airway relevance following radiotherapy
Source: Clin Transl Radiat Oncol. 2026 Jul 29;60:101248. doi: 10.1016/j.ctro.2026.101248 (PMC13452148; doi:10.1016/j.ctro.2026.101248)
Supplement: Supplementary file 1 [file mmc1.docx]

**Supplementary Table A.** Comparison by anatomical site (Other vs. Oropharynx).

| **Variable** | **Other (n = 24)** | **Oropharynx (n = 34)** | ***p*** | |
| --- | --- | --- | --- | --- |
| **Sociodemographic** | | | |  |
| Age (years), mean ± SD | 54.2 ± 14.8 | 58.2 ± 9.0 | 0.239ᵃ | |
| Female sex, n (%) | 5 (20.8) | 7 (20.6) | 1.000^b^ | |
| BMI (kg/m²), mean ± SD | 25.9 ± 5.6 | 26.2 ± 5.6 | 0.849 | |
| **Sleep-disordered breathing** | | | |  |
| STOP-Bang high risk, n/N (%) | 2/23 (8.7) | 10/33 (30.3) | 0.096^b^ | |
| Loud snoring, n/N (%) | 4/16 (25.0) | 8/24 (33.3) | 0.729^b^ | |
| **Sleep quality** | | | |  |
| ESS total score, mean ± SD | 8.54 ± 4.86 | 8.12 ± 4.42 | 0.731 | |
| ISI total score, mean ± SD | 9.21 ± 6.54 | 10.06 ± 6.15 | 0.615 | |
| PSQI total score, mean ± SD | 7.75 ± 3.73 | 8.32 ± 4.04 | 0.585 | |
| Poor sleep quality (PSQI > 5), n/N (%) | 14/24 (58.3) | 26/34 (76.5) | 0.162^b^ | |
| **Fatigue** | | | |  |
| Chalder Fatigue Scale, mean ± SD | 17.2 ± 4.9 | 19.0 ± 5.4 | 0.199 | |
| Elevated fatigue (CFS ≥ 18), n/N (%) | 11/24 (45.8) | 19/34 (55.9) | 0.595^b^ | |
| **Psychological distress** | | | |  |
| HADS-Anxiety, mean ± SD | 5.91 ± 3.44 | 5.56 ± 3.28 | 0.710 | |
| HADS-Depression, mean ± SD | 4.68 ± 3.55 | 5.19 ± 3.84 | 0.626 | |
| **Health-related quality of life** | | | |  |
| SF-12 PCS, mean ± SD | 46.9 ± 7.9 | 44.6 ± 10.6 | 0.398 | |
| SF-12 MCS, mean ± SD | 42.5 ± 9.3 | 44.2 ± 8.8 | 0.491 | |

BMI = body mass index; ESS = Epworth Sleepiness Scale; HADS = Hospital Anxiety and Depression Scale; ISI = Insomnia Severity Index; MCS = Mental Component Summary; PCS = Physical Component Summary; PSQI = Pittsburgh Sleep Quality Index; SF-12 = Short Form-12.

All p-values are two-sided; α = 0.05. Independent-samples t-tests for continuous outcomes; Welch's correction (ᵃ) applied where Levene's test indicated unequal variances. Fisher's Exact test (^b^) used for all categorical comparisons.

**Supplementary Table B.** Comparison by airway impact (Low vs. High).

| **Variable** | **Low impact**  **(n = 18)** | **High impact**  **(n = 40)** | ***p*** |
| --- | --- | --- | --- |
| **Sociodemographic** | | | |
| Age (years), mean ± SD | 53.7 ± 14.5 | 57.8 ± 10.3 | 0.224 |
| Female sex, n (%) | 3 (16.7) | 9 (22.5) | 0.736^b^ |
| BMI (kg/m²), mean ± SD | 27.7 ± 7.2 | 25.3 ± 4.5 | 0.204ᵃ |
| **Sleep-disordered breathing** | | | |
| STOP-Bang high risk, n/N (%) | 5/18 (27.8) | 7/38 (18.4) | 0.494^b^ |
| Loud snoring, n/N (%) | 2/13 (15.4) | 10/27 (37.0) | 0.271^b^ |
| **Sleep quality** | | | |
| ESS total score, mean ± SD | 9.06 ± 4.08 | 7.95 ± 4.79 | 0.399 |
| ISI total score, mean ± SD | 8.39 ± 4.35 | 10.30 ± 6.94 | 0.287 |
| PSQI total score, mean ± SD | 7.39 ± 3.01 | 8.40 ± 4.22 | 0.364 |
| Poor sleep quality (PSQI > 5), n/N (%) | 11/18 (61.1) | 29/40 (72.5) | 0.540^b^ |
| **Fatigue** | | | |
| Chalder Fatigue Scale, mean ± SD | 17.2 ± 3.9 | 18.8 ± 5.7 | 0.224ᵃ |
| Elevated fatigue (CFS ≥ 18), n/N (%) | 11/18 (61.1) | 19/40 (47.5) | 0.402^b^ |
| **Psychological distress** | | | |
| HADS-Anxiety, mean ± SD | 4.63 ± 2.94 | 6.16 ± 3.40 | 0.122 |
| HADS-Depression, mean ± SD | 3.50 ± 3.56 | 5.61 ± 3.62 | 0.055 |
| **Health-related quality of life** | | | |
| SF-12 PCS, mean ± SD | 46.7 ± 8.2 | 45.0 ± 10.2 | 0.549 |
| SF-12 MCS, mean ± SD | 46.0 ± 8.3 | 42.5 ± 9.2 | 0.187 |

BMI = body mass index; ESS = Epworth Sleepiness Scale; HADS = Hospital Anxiety and Depression Scale; ISI = Insomnia Severity Index; MCS = Mental Component Summary; PCS = Physical Component Summary; PSQI = Pittsburgh Sleep Quality Index; SF-12 = Short Form-12.

All p-values are two-sided; α = 0.05. Independent-samples t-tests for continuous outcomes; Welch's correction (ᵃ) applied where Levene's test indicated unequal variances. Fisher's Exact test (^b^) used for all categorical comparisons.

**Supplementary Table C.** Comparison by chemotherapy status (No vs. Yes).

| **Variable** | **No chemotherapy (n = 29)** | **Chemotherapy**  **(n = 29)** | ***p*** |
| --- | --- | --- | --- |
| **Sociodemographic** | | | |
| Age (years), mean ± SD | 57.1 ± 13.3 | 56.0 ± 10.3 | 0.725 |
| Female sex, n (%) | 7 (24.1) | 5 (17.2) | 0.747^b^ |
| BMI (kg/m²), mean ± SD | 25.5 ± 4.7 | 26.8 ± 6.3 | 0.380 |
| **Sleep-disordered breathing** | | | |
| STOP-Bang high risk, n/N (%) | 6/29 (20.7) | 6/27 (22.2) | 1.000^b^ |
| Loud snoring, n/N (%) | 8/22 (36.4) | 4/18 (22.2) | 0.491^b^ |
| **Sleep quality** | | | |
| ESS total score, mean ± SD | 8.24 ± 4.87 | 8.34 ± 4.35 | 0.932 |
| ISI total score, mean ± SD | 10.24 ± 5.71 | 9.17 ± 6.85 | 0.521 |
| PSQI total score, mean ± SD | 8.10 ± 3.64 | 8.07 ± 4.19 | 0.973 |
| Poor sleep quality (PSQI > 5), n/N (%) | 19/29 (65.5) | 21/29 (72.4) | 0.777^b^ |
| **Fatigue** | | | |
| Chalder Fatigue Scale, mean ± SD | 18.1 ± 5.4 | 18.4 ± 5.1 | 0.861 |
| Elevated fatigue (CFS ≥ 18), n/N (%) | 14/29 (48.3) | 16/29 (55.2) | 0.793^b^ |
| **Psychological distress** | | | |
| HADS-Anxiety, mean ± SD | 5.63 ± 3.14 | 5.78 ± 3.55 | 0.871 |
| HADS-Depression, mean ± SD | 4.26 ± 3.01 | 5.70 ± 4.21 | 0.154ᵃ |
| **Health-related quality of life** | | | |
| SF-12 PCS, mean ± SD | 46.4 ± 8.8 | 44.7 ± 10.5 | 0.512 |
| SF-12 MCS, mean ± SD | 44.3 ± 7.9 | 42.8 ± 10.1 | 0.525 |

BMI = body mass index; ESS = Epworth Sleepiness Scale; HADS = Hospital Anxiety and Depression Scale; ISI = Insomnia Severity Index; MCS = Mental Component Summary; PCS = Physical Component Summary; PSQI = Pittsburgh Sleep Quality Index; SF-12 = Short Form-12.

All p-values are two-sided; α = 0.05. Independent-samples t-tests for continuous outcomes; Welch's correction (ᵃ) applied where Levene's test indicated unequal variances. Fisher's Exact test (^b^) used for all categorical comparisons.
